# Community Environments That Promote Intergenerational Interactions vs. Walking Among Older Adults

**DOI:** 10.3389/fpubh.2020.587363

**Published:** 2020-12-03

**Authors:** Sinan Zhong, Chanam Lee, Hanwool Lee

**Affiliations:** Department of Landscape Architecture and Urban Planning, College of Architecture, Texas A&M University, College Station, TX, United States

**Keywords:** intergenerational communities, interactions with children, intergenerational interactions, transportation walking, recreational walking, older adults

## Abstract

**Background:** Intergenerational interactions and walking are two of the most beneficial forms of activities for older adults. As older adults spend most of their time at or near home, the characteristics of the proximate residential environments are particularly important for supporting those activities. This study aims to (1) explore places used for various social interactions older adults engage in, (2) examine specific neighborhood environmental features associated with intergenerational interactions, and (3) compare similarities and differences in environmental correlates of intergenerational interactions vs. walking.

**Methods:** This cross-sectional study analyzed self-reported survey data from 455 community-dwelling adults aged 65+ from Austin, Texas, as well as Geographic Information System (GIS) measures capturing the neighborhood environment around each participant's home. Descriptive statistics were used for Aim 1. Multivariable binary logistic models were used for Aims 2 and 3, to identify environmental variables predicting the odds of participating in intergenerational interactions (with children 1+ times/week, and with children, teenagers, or adults 1+ times/week) in one's neighborhood, as well as walking 1+ times/week for transportation or recreation purposes.

**Results:** Participants had a mean age of 73 years, and were primarily female (72.1%) and non-Hispanic white (72.8%). Older adults interacted frequently with adults (79.2%, 1+ times/week) and other older adults (66.9%) in their neighborhood, while less frequently with children (28.0%) and teenagers (21.9%). Recreational walking (73.3%, 1+ times/week) was more popular for older adults than transportation walking (43.8%). Multivariable analyses showed that neighborhood perceptions, transportation infrastructure, land uses, land covers, population densities, development activities, and composite scores were significant predictors of intergenerational activities. Both similarities and differences were found in terms of the neighborhood environmental factors associated with intergenerational interactions vs. walking although differences were more evident in the domains of land covers, development activities, and composite scores.

**Conclusions:** Given the significant health benefits, promoting intergenerational interactions and walking among older adults should be a national/global responsibility. Further work is needed to improve our understanding of the specific social and physical environmental facilitators as well as barriers to creating intergenerational communities that can support healthy living of all generations.

## Introduction

Demographic aging is a global issue that can bring tremendous economic, social, and medical challenges. The United States (US) population aged 65 years and over more than tripled from 13.0 million in 1950 to 53.3 million in 2019 and is expected to increase to 84.8 million in 2050 (1). Ageism, which is defined as negative stereotypes based on age, is another challenge that the aging society faces. Ageism originates from a fear of being older, a shortage of knowledge about aging, and limited interactions with the elderly, which can result in serious adverse effects on older adults (2). Engaging in intergenerational activities is increasingly recognized as a promising means to reduce ageism and social isolation while also promoting active and healthy lifestyles in old age. As older adults spend most of their time at home and in their neighborhood, understanding the relationships between neighborhood environments and older adults' intergenerational interactions is critical to creating/retrofitting neighborhood environments that can support active and healthy aging in place.

Increasing empirical investigations indicate the significant roles of intergenerational interactions in maintaining older adults' health. Specifically, a number of program-based intergenerational activities have been shown to be positively correlated with older adults' physical health (3–8), psychosocial health (e.g., reduced depression) (7, 9–14), self-reported quality of life/well-being (15, 16), and social relationships (e.g., reduced social isolation) (6, 17, 18). Additionally, participation in intergenerational programs has been linked with physical activity (6, 19–24) and social activity (19) among older adults.

Physical activity is another major factor that can contribute to promoting and maintaining health in aging populations (25). Walking is one of the most popular and accessible forms of physical activity among older adults, even though there are a variety of ways to stay physically active (26, 27). The significant health benefits of walking for aging populations have been well-documented in many empirical studies. Hakim et al. (28) reported that regular walking was linked with lower mortality rates among non-smoking retired men. Moreover, several studies on walking and depression demonstrated positive associations between walking and reduced depressive symptoms among older adults (26, 29, 30).

Despite its significant health benefits, most older adults do not engage in sufficient amounts of physical activity. According to the 2018 Behavioral Risk Factor Surveillance System data (31), ~30.3% of the US population aged 65 years and over reported no physical activity other than those done as part of work/jobs. The prevalence of physical inactivity among the US older populations increases significantly with age. Approximately 35.1% of the US populations aged 75 years and over reported no leisure time physical activity compared to 26.9% among those aged 65–74 years in 2018 (31). The high prevalence of inactivity among older adults in the US has brought attention to the need for broader environmentally-based approaches to facilitate population level behavioral changes.

According to M. Powell Lawton's influential work on environments and aging (32), our environments (e.g., personal, social, and physical environments) play essential roles in promoting older adults' health. Many empirical studies have evinced that neighborhood environments (e.g., walkability) are associated with older adults' physical activity including walking (33, 34). Evidence has also been accumulating about the significant roles of neighborhood environments in maintaining older adults' physical health (35, 36), mental health (37), and quality of life (38, 39). However, limited studies have investigated the associations between neighborhood environments and intergenerational interactions among older adults. Only a small number of empirical studies have reported significant correlations between neighborhood environments (e.g., walkability, accessibility) and older adults' social interactions/participations (40, 41). These studies have considered overall social activities, without fully addressing the influences of neighborhood environments on older adults' intergenerational interactions.

This study aims to (1) explore places used for various social interactions older adults engage in, (2) examine specific neighborhood environmental features associated with older adults' intergenerational interactions, and (3) compare similarities and differences in environmental correlates of intergenerational interactions vs. walking. Going beyond the scope of existing empirical studies on environments and aging, this study provides a systematic examination of physical elements/features of the community environment that can promote intergenerational interactions and/or walking.

## Materials and Methods

### Conceptual Framework

**Figure 1:**
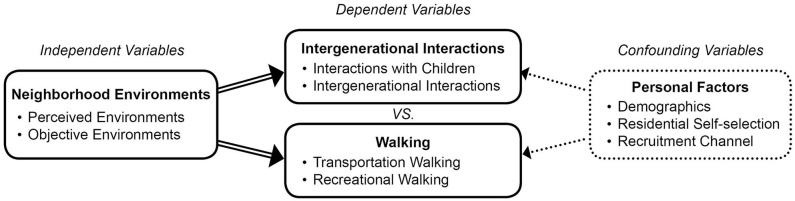
Conceptual framework for environmental correlates of intergenerational interactions vs. walking among older adults.

Lawton's seminal work on environments and aging, the social ecological model of health promotion (42), and prior literature on this topic as described above point to personal and environmental factors as major determinants of older adults' intergenerational interactions and walking. Figure 1 shows a conceptual framework with the hypothesized relationships among neighborhood environments (i.e., perceived and objectively measured physical environments), intergenerational interactions (i.e., social interactions with children, intergenerational interactions), walking (i.e., transportation and recreational walking), and personal factors (i.e., demographics, residential self-selection, recruitment channel) among older adults. The conceptual framework is developed to guide the data collection and analysis process for achieving the three research aims and answering the following research questions:

- How can neighborhood environments contribute to promoting or inhibiting older adults' intergenerational interactions?
- What differential roles do neighborhood environments play in older adults' intergenerational interactions vs. walking?

### Study Setting and Population

This cross-sectional study was carried out in the city of Austin, Texas, US, which has a wide range of services and programs supporting older adults, diverse environmental characteristics, and a diverse mix of different age groups. The target population is community-dwelling Austin residents who are 65 years and older. The age limit of 65 years is a commonly used threshold for defining older adults in the US (43). Although Austin had a relatively lower percentage of older residents 65 years and older (9.4%) compared to Texas (12.0%) and the US (15.2%) as of 2018 (44), its aging population was growing at a rate (85.2%) much faster than Texas (61.1%) and the US (40.7%) from 2000 to 2018 (44, 45).

### Recruitment and Data Collection

Data for this study included both subjective measures of self-report surveys and objective measures from Geographic Information System (GIS) and Walk Score (walkscore.com) capturing the neighborhood environment around each survey participant's home. The survey was offered both online and in paper form, took ~30 minutes to complete, and captured variables related to (1) physical activities and walking, (2) quality of life and mental health (i.e., depression), (3) intergenerational and other social activities, (4) neighborhood environments, (5) supportive services or programs, and (6) demographic and socioeconomic characteristics. The survey was available in English only, as the majority (~91.8%) of Austin residents 65 years and over reported sufficient English proficiency (i.e., speak only English, speak English very well, and speak English well) (44).

The survey development and data collection processes were carried out in four phases, starting from a three-phase process to develop and test the preliminary and final survey instrument, which was critical to ensure the validity and reliability of the data collected for this study. The process included (1) a pilot study to solicit input on the design and content of the preliminary paper survey through focus groups (Phase 1: May–June 2018); (2) a pre-test of the preliminary online and paper survey among a small number of participants (Phase 2: August–October 2018); and (3) a test-retest reliability assessment of the final survey instrument (Phase 3: January 2019–June 2019). The last phase (Phase 4: October 2018–June 2019) involved actual data collection using the finalized survey instrument. All study protocols and materials were approved by the Texas A&M University Institutional Review Board.

**Figure 2:**
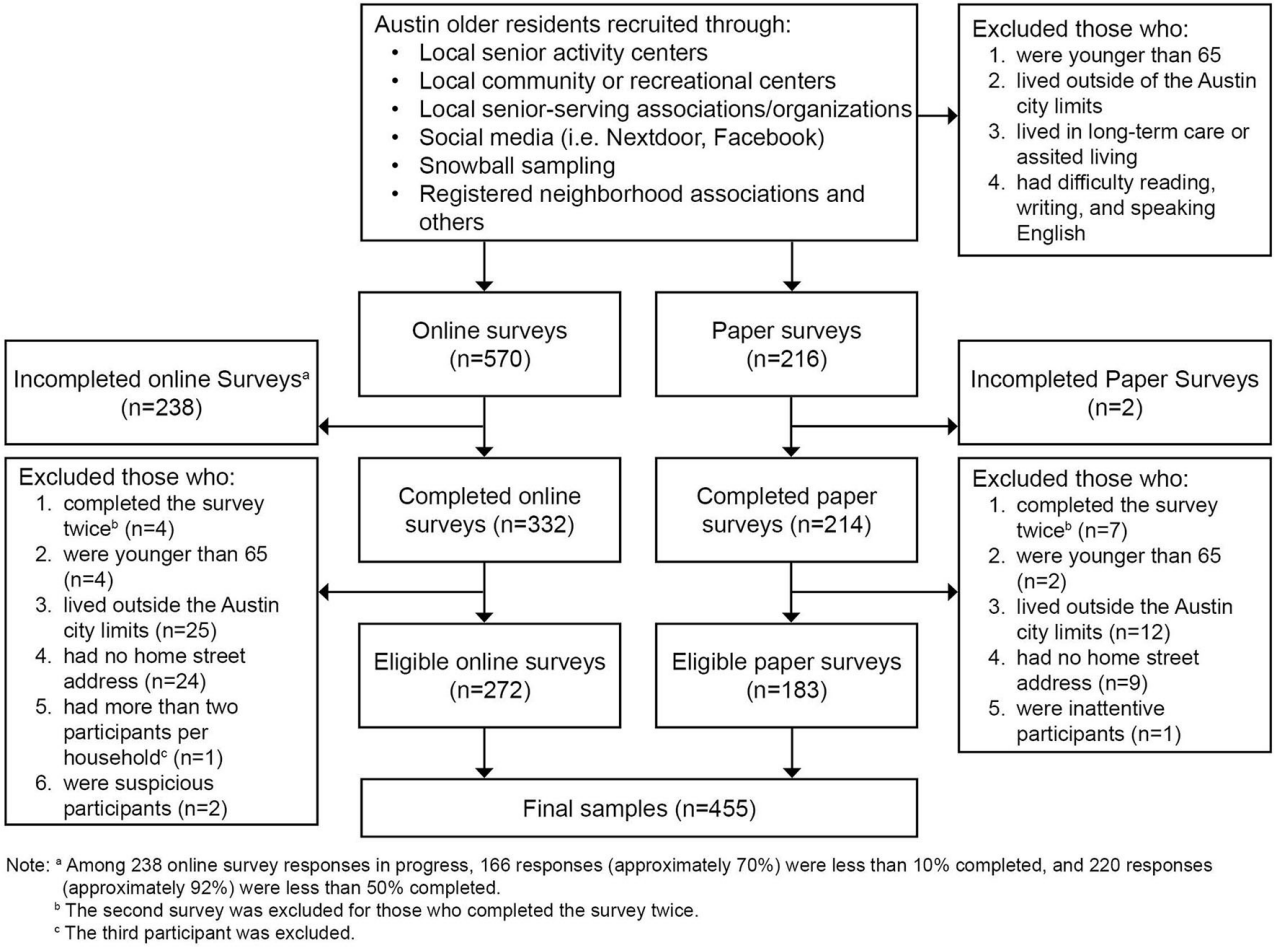
Survey data collection and screening process.

Figure 2 summarizes the detailed steps during the Phase 4 survey data collection and screening process. After excluding 91 respondents who failed to meet the eligibility criteria, a total of 455 eligible older adult respondents completed the survey, containing 272 online and 183 paper surveys. To be eligible, the respondents had to be the residents of Austin, Texas who (1) are 65 years or older, (2) live in the ordinary communities instead of long-term care or assisted living facilities, and (3) have basic English language skills. Up to two eligible participants per household could join the survey. Convenience sampling strategy was used due to the typically low response and eligibility rates expected from random sampling for studies like this and due to the limited resources available for this study. Participant characteristics were closely monitored throughout the survey process to ensure adequate spatial and sociodemographic diversity and representativeness of the samples.

Recruitment efforts targeted the senior participants/members at local senior-serving centers [number of participants (*n*) = 225, 49.6%]. These centers included (a) three senior activity centers containing Lamar (*n* = 54, 11.9%), South Austin (*n* = 55, 12.1%), and Conley Guerrero senior activity centers (*n* = 46, 10.1%); and several community or recreational centers (*n* = 28, 6.2%) managed by the City of Austin Parks and Recreation Department, (b) WellMed Charitable Foundation Senior Community Center (*n* = 45, 9.9%), and (c) Oak Hill Senior Center managed by the Meals on Wheels (*n* = 6, 1.3%). Study flyers were also distributed by various local senior-serving associations/organizations (*n* = 40, 8.8%), including AustinUp, Aging is Cool, Aging2.0 Austin, American Association of Retired Persons, AGE of Central Texas, Capital City Village, and Austin Retired Teachers Association. Additionally, social media (i.e., Nextdoor and Facebook) were utilized to recruit 78 study participants (17.2%), and a snowball sampling was applied to recruit 79 study participants (17.4%) by asking existing participants to share the study information with their families, friends, and neighbors. Finally, 38 more study participants (8.4%) were recruited from registered neighborhood associations, churches, and community gardens in Austin, Texas. Eighteen participants indicated that they learned about our study from more than one source.

### Measures

#### Dependent Variables

##### Intergenerational Interactions

No validated instruments were available to capture intergenerational and other social activities in one's neighborhood (46). Therefore, relevant items were newly developed for this study, after several rounds of pilot studies/pretests as described earlier. The finalized survey question, “in your neighborhood, how many days in a typical week do you spend at least 10 minutes interacting (talking, spending time together) with others of different ages?” was used to measure study participants' social interactions with children, teenagers, adults, and older adults, separately. After checking the distribution of the original data, two binary outcome variables were generated to capture older adults' intergenerational interactions: interacting with children 1+ times/week in one's neighborhood, and interacting with children, teenagers, or adults 1+ times/week in one's neighborhood.

##### Walking

Walking for transportation (e.g., walking to get to and from places) and recreation (e.g., walking for recreation, sport, exercise, or leisure) were captured by four survey questions adapted from the International Physical Activity Questionnaires (47). We used two questions: “in a typical week, how many days do you walk for transportation (for recreation)?' and “how much time do you usually spend walking for transportation (for recreation) on one of those days?” to measure each of the two walking types. Transportation walking and recreation walking were recoded as two binary variables (i.e., walking 1+ times/week vs. not for recreation/transportation) as a considerable proportion of the study participants reported not walking for transportation (56.2%) or recreation (26.7%) in a typical week.

#### Independent Variables: Neighborhood Environments

##### Perceived Physical Environments

The survey questions evaluating neighborhood environments were extracted or adapted primarily from the Neighborhood Environment Walkability Scale (48, 49). Residence in a newly built neighborhood was measured through a multiple-choice question, “do you currently live in…?” that included a response item of “newly built neighborhood (built in the last 10–15 years).” One statement on a four-point Likert scale from strongly disagree to strongly agree, “there are benches on most of the sidewalks in my neighborhood,” was used to measure the availability of benches, which was dichotomized as strongly disagree vs. others because of its uneven distribution.

Three more latent factor variables, including neighborhood walkability, neighborhood aesthetics, and traffic safety, were generated by conducting the principal component analysis with the Promax oblique rotation among the neighborhood environment survey items captured on a four-point Likert scale from strongly disagree to strongly agree. Specifically, the neighborhood walkability factor captured four survey items: “stores are within easy walking distance of my home,” “there are many places to go within easy walking distance of my home,” “it is easy to walk to a transit stop (bus, train) from my home,” and “it is easy to walk to healthcare/medical services (e.g., hospital, doctor's office, pharmacy).” Another four survey items were used to extract the neighborhood aesthetics factor: “there are many interesting things to look at while walking in my neighborhood,” “my neighborhood is generally free from litter,” “there are many attractive natural sights in my neighborhood (such as landscaping, views),” and “there are attractive buildings/homes in my neighborhood.” The traffic safety latent factor contained four reversed coding survey items: “there is so much traffic along the street I live on that it makes it difficult or unpleasant to walk in my neighborhood,” “there is so much traffic along nearby streets that it makes it difficult or unpleasant to walk in my neighborhood,” “when walking in my neighborhood, there are a lot of exhaust fumes (such as from cars, buses),” and “most drivers exceed the posted speed limits while driving in my neighborhood.”

##### Objective Physical Environments

We examined five domains of objective physical environments, including (1) transportation, (2) land uses, (3) land covers, (4) population densities and development activities, and (5) composite scores, which were selected based on the previous literature on environment-walking and environment-social interaction relationships (33, 34, 40, 50). The first four domains were measured through GIS variables captured within a ½-mile buffer around the participants' homes and as the shortest network distances. This study incorporated two types of ½-mile buffers, including airline and street network-based “sausage” buffers. The sausage buffer referred to buffering all streets located within a ½-mile street distance from each participant's home, and for a “radius” of 100 feet on both sides of the street center line (51–53). This buffer is superior to airline or standard street network buffers in that it better estimates the street environment that pedestrians are actually exposed to. Most of the GIS variables were captured within the ½-mile sausage buffer, covering the domains of transportation infrastructure, general and destination land uses, and land covers (i.e., area of tree canopies). Additionally, the ½-mile airline buffer was used to capture several additional attributes related to parks, water bodies, development permits, and population densities, which tend to be more sensitive to the dependent variables when captured within the larger airline buffer (54, 55).

The raw data for the GIS measures used in this study were collected as part of the Active Living Austin research project sponsored by NIH (R01CA197761). Most of the raw data were downloaded from the Austin Open Data Portal (data.austintexas.gov), including 2019 data for street segments, sidewalks, general land uses, and development permits, as well as 2016 water body data. Destination land use data for retail/services, institutional, sports and fitness, and undesirable destinations were downloaded from the ESRI business analyst webpage (bao.arcgis.com) in 2019. Public transit data regarding transit stops and transit routes were downloaded from the Capital Area Metropolitan Planning Organization (data.texas.gov) in 2019. The stop sign and park-related data were collected from the Austin Transportation Department in 2017 and the Austin Park and Recreation Department in 2019, respectively. Tree canopies were calculated based on the 2016 Texas NAIP Imagery data downloaded from the Texas Natural Resources Information System (data.tnris.org). The street intersection variables were calculated based on the street segment layer. Street intersections with stop signs were calculated if the distance between each intersection and its closest stop sign was <50 feet. The population density variable was calculated based on the 2018 census block group population data (44) using the following formula: POP_*density*_ $=\overset{n}{\underset{i=1}{\sum }}NiPi/A$, where POP_*density*_ is the population density within the ½-mile airline buffer; Ni is the number of people within each census block group; Pi is the percentage of the residential land use located within the ½-mile airline buffer for each census block group; n is the total number of census block groups within the ½-mile airline buffer; and A is the area of the residential land use within the ½-mile airline buffer or the area of the ½-mile airline buffer for calculating the net or gross population density, respectively.

In addition to these detailed disaggregated measures, widely available aggregated measures including Walk Score, Transit Score, and Bike Score were collected through the 2019 Walk Score (walkscore.com) and examined as supplementary variables in this study. Empirical studies investigated that these composite scores served as validated measures of overall neighborhood walkability (56, 57) and for considering mobility and walking among older adults (50, 58).

#### Confounding Variables

##### Demographics

All survey questions measuring participants' demographics and socioeconomic characteristics were extracted or adapted from the Behavioral Risk Factor Surveillance System (59); the American Community Survey (60); two survey instruments developed by the AdvantAge Initiative, Center for Home Care Policy & Research, Visiting Nurse Service of New York; and the Neighborhood Quality of Life Survey for Seniors (33). Seven variables were included in all regression models: age (years), gender (male vs. female), race and ethnicity (non-Hispanic White vs. others), marital status (married or unmarried couples vs. others), education attachment (nine levels from less than high school to doctorate degree), income (i.e., low, lower-middle, upper-middle, high, don't know/prefer not to answer/missing), and general health conditions (i.e., excellent, very good, good, fair, poor). Another seven variables that were included in at least one model included: housing types (one-family detached house vs. others), having a dog in the household (yes vs. no), employment status (employed vs. not employed), daily sleep time (hours), difficulty walking (yes/don't know/prefer not to answer vs. no), mobility aids (yes vs. no), and the significant life event regarding personal illness (yes vs. no). Other variables that were tested but insignificant in the multivariable regression analyses included body mass index, home ownership, having a cat in the household, living arrangement (except living with a spouse that was highly correlated with marital status), caregiving status, diseases (e.g., anxiety, depression, cancer), difficulty hearing or seeing, alcoholic consumption, history of falls, and significant life events (i.e., illness of a family member or friend, death of a spouse, family member, or friend, non-medical events).

##### Residential Self-Selection

Residential self-selection factors are important to help address the self-selection bias inherent in cross-sectional studies like this (61). In this research, those factors were measured by asking participants to rate the importance of a series of reasons behind their residential location choice: “how important are the following reasons for you to choose living in your current home?” with a four-point Likert response option (i.e., not at all important, slightly important, moderately important, very important). The variable capturing the diversity of age groups in the neighborhood was retained as an important individual variable for this study focusing on intergenerational activities, instead of entering into the factor analysis. The diversity of ethnic groups was excluded due to its high correlation with the diversity of age groups (*r* = 0.798). Another two variables were dichotomized and considered as individual variables because they fail to be properly loaded to a single latent factor: affordability (very important vs. others) and proximity to public transportation (not at all important vs. others). Affordability was not included in this study as it had no significant associations with any of the outcomes.

The principal component analysis with the Promax oblique rotation was conducted with the remaining twelve items to generate two latent factor variables measuring participants' self-selection on neighborhood environments and neighborhood social cohesions. The neighborhood environment self-selection factor was loaded with eight survey items: “ease of walking,” “neighborhood aesthetics or beautiful scenery,” “sense of community,” “close to parks and natural open spaces,” “neighborhood safety,” “close to shops and services,” “close to healthcare/medical facilities,” and “close to entertainment facilities.” Another four survey items were included to measure their residential self-selection based on neighborhood social cohesions: “close to friends,” “presence of other older residents,” “access to supportive programs,” and “close to family members.”

##### Recruitment Channel

Participants were asked to indicate how they learned about the study on a multiple-choice question: “how did you hear about this study?” with options for different recruitment channels used in this study. Each recruitment option was converted into a binary variable and tested in the multivariable regression models. Only one variable, social media recruitment (yes vs. no), was significant and controlled for recreational walking.

### Statistical Analyses

This study used IBM SPSS Statistics 25.0 to generate all descriptive and inferential statistics. Descriptive statistics, including central tendency, dispersion or variation, and distribution, were examined to understand the basic features of the study variables. Bivariate analyses (i.e., independent samples *t*-test, chi-square test) were conducted between the independent/confounding variables and each of the four outcome measures (results not reported).

Multivariable binary logistic regressions were estimated in two steps to identify significant (*p* < 0.05) correlates of intergenerational interactions and walking among older adults. The first step was to build a base model for each of the four outcomes by regressing individual intergenerational interaction or walking variable on significant demographic/socioeconomic, residential self-selection, and recruitment channel variables (confounding variables) identified in the previous bivariate analyses. The second step was to conduct one-by-one tests where the physical environmental variables (independent variables) were added to the base models one at a time. Because many of the physical environmental variables were strongly associated with each other, this one-by-one testing approach helped examine the statistical significance of each independent variable without the impact of other correlated variables, to guide the selection of optimal variables for further consideration in the final multivariable model. The Variance Inflation Factors (VIFs) were examined to assess the potential for multicollinearity problems in all multivariable models, and the values ranged from 1.0 to 1.6 suggesting low/minimal risks.

## Results

### Participant Characteristics

Table 1 summarizes the study characteristics in terms of personal factors, intergenerational interactions and walking, and neighborhood environments. The age range was 65 to 95, with a mean age of 73. Participants were about 72.1% female, 72.8% Non-hispanic white, and 41.7% married. Approximately 85.7% of respondents had at least some college education. As for the general health conditions, the majority (86.4%) reported their health to be good, very good, or excellent.

**Table 1:** Study characteristics.

**Variable**	***N***	**Mean/Freq (SD/%) Min-Max**	**Variable**	***N***	**Mean/Freq (SD/%) Min-Max**
**DEMOGRAPHICS**					
Age (years)	455	73.06 (6.19)	General health condition: Poor	449	7 (1.6%)
		65-95	Fair		54 (12.0%)
Gender: Male	455	127 (27.9%)	Good		159 (35.4%)
Female		328 (72.1%)	Very Good		160 (35.6%)
Race and ethnicity: Non-Hispanic White	452	329 (72.8%)	Excellent		69 (15.4%)
Others		123 (27.2%)	Income: Low income (below $20,000)	455	65 (14.3%)
Marital status: Married or unmarried couple	453	205 (45.3%)	Lower-middle income ($20,000-$39,999)		86 (18.9%)
Others		248 (54.7%)	Upper-middle income ($40,000-$79,999)		125 (27.5%)
Education: Less than high school	455	8 (1.8%)	High income ($80,000 or more)		99 (21.8%)
Some high school, but no degree		12 (2.6%)	Don't know/prefer not to answer/missing		80 (17.6%)
High school diploma/GED		45 (9.9%)	Having a dog in the household: Yes	455	113 (24.8%)
Some college		64 (14.1%)	No (or missing)		342 (75.2%)
Associate degree		27 (5.9%)	Mobility aid: Yes	441	56 (12.7%)
Bachelor's degree		122 (26.8%)	No		385 (87.3%)
Master's degree		110 (24.2%)	Personal illness: Yes	448	192 (42.9%)
Professional degree		25 (5.5%)	No		256 (57.1%)
Doctorate degree		42 (9.2%)	Daily sleep time (hours)	444	7.25 (1.36)
Employment status: Employed	455	82 (18.0%)			2–16
Not employed		373 (82.0%)	Difficulty walking: Yes/don't know/prefer not to answer	453	103 (22.7%)
Housing type: One-family detached house	455	344 (75.6%)	No		350 (77.3%)
Others		111 (24.4%)			
**RESIDENTIAL SELF-SELECTION AND RECRUITMENT CHANNEL**					
Diversity of age groups: Not at all important	455	115 (25.3%)	Neighborhood environments (factor scores)	455	0.00 (0.99)
Slightly important		106 (23.3%)			−2.71–1.54
Moderately important		144 (31.6%)	Close to public transportation: Not at all important	455	172 (37.8%)
Very important		90 (19.8%)	Others		283 (62.2%)
Social cohesion and support (factor scores)	455	0.00 (0.99)	Social media (i.e., Nextdoor, Facebook): Yes	454	78 (17.2%)
		−1.74–2.40	No		376 (82.8%)
**INTERGENERATIONAL INTERACTIONS AND WALKING**					
Social interactions with children: Yes	453	127 (28.0%)	Transportation walking: Yes	441	193 (43.8%)
No		326 (72.0%)	No		248 (56.2%)
Intergenerational interactions: Yes	453	363 (80.1%)	Recreational walking: Yes	442	324 (73.3%)
No		90 (19.9%)	No		118 (26.7%)
**PERCEIVED PHYSICAL ENVIRONMENTS**					
Newly built neighborhood: Yes	455	50 (11.0%)	Neighborhood aesthetics (factor scores)	455	0.00 (1.00)
No (or missing)		405 (89.0%)			−3.22–1.31
Neighborhood walkability (factor scores)	455	0.00 (1.00)	Traffic safety (factor scores)	455	0.00 (1.00)
		−1.50–1.94			−2.47–1.61
Benches on most of the sidewalks^a^: Yes	455	149 (32.7%)			
No		306 (67.3%)			
**OBJECTIVE PHYSICAL ENVIRONMENTS (SAUSAGE BUFFER)**					
***Transportation***			***Transportation***		
Street length (miles)	453	6.69 (3.01)	Number of Intersections with 3 or more ways (n)	453	44.77 (25.01)
		0.36–15.53			0–129
Sidewalk length (miles)	453	11.04 (5.08)	Density of intersections with 3 or more ways (n/acre)	453	6.43 (1.39)
		0.24–24.93			0–9.55
Length of high–speed streets (>30 mph) (miles)	453	2.79 (1.67)	***Land Uses***		
		0.00–11.17	Area of offices (acres): 0	453	140 (30.9%)
Percentage of high-speed streets	453	42.2% (17.8%)	>0– <1.5		155 (34.2%)
(>30 mph)		0.0–100.0%	≥1.5		158 (34.9%)
Number of transit stops (n): 0	453	105 (23.2%)	Percentage of offices: 0%	453	140 (30.9%)
1–5		121 (26.7%)	>0% – <2%		210 (46.4%)
6–10		100 (22.1%)	≥2%		103 (22.7%)
11 or more		127 (28.0%)	Presence of food stores: Yes	453	160 (35.3%)
Density of transit stops (n/100 acres):	453		No		293 (64.7%)
Lower density: 0 – <10		349 (77.0%)	Presence of religious destinations: Yes	453	195 (43.0%)
Higher density: ≥10		104 (23.0%)	No		258 (57.0%)
Number of total transit routes (n)	453	3.80 (4.43)	Presence of trails in parks: Yes	453	161 (35.5%)
		0–35	No		292 (64.5%)
Number of stop signs (n)	453	45.53 (35.09)	Presence of sports and fitness destinations: Yes	453	115 (25.4%)
		0–184	No		338 (74.6%)
Density of stop signs (n/acre)	453	6.14 (2.58)	Presence of locally undesirable destinations: Yes	453	203 (44.8%)
		0–15.02	No		250 (55.2%)
Number of intersections with stop signs (n)	453	25.53 (17.99)	***Land Covers***		
		0–97	Area of tree canopies (acres)	453	42.46 (23.48)
Percentage of intersections with stop signs	453	53.5% (19.9%)			2.37–122.42
		0.0–92.9%			
**OBJECTIVE PHYSICAL ENVIRONMENTS (AIRLINE BUFFER)**					
***Land Uses***			***Land Covers***		
Presence of greenbelts: Yes	453	191 (42.2%)	Presence of water bodies: Yes	453	53 (11.7%)
No		262 (57.8%)	No		400 (88.3%)
Number of parks, excluding natural	453	2.35 (1.97)	***Development Activities***		
preserved and greenbelt types (n)		0–11	Number of all development permits issued in 2019 [ln(n)]	442	3.80 (1.50)
***Population Densities***					0.00–6.74
Net population density (n/acre)	453	18.31 (9.32)	Commercial permits issued in 2019: Yes	453	199 (43.9%)
		2.84–82.68	No		254 (56.1%)
Gross population density (n/acre)	453	8.24 (3.69)	Residential permits issued in 2019: Yes	453	345 (76.2%)
		1.17–28.36	No		108 (23.8%)
**OBJECTIVE PHYSICAL ENVIRONMENTS (SHORTEST NETWORK DISTANCE)**					
***Transportation***			***Land Uses***		
Proximity to the closest transit stop [ln(miles)]	450	−1.36 (1.16)	Proximity to the closest food store (miles)	455	0.65 (0.50)
		−8.72–1.60			0.00–5.11
Proximity to the closest rail station [ln(miles)]	452	1.26 (0.77)	Proximity to the closest park with/next to a water	452	0.81 (1.27)
		−2.59–2.83	body [ln(miles)]		−5.07–2.71
Transit routes at the closest stop (n): 1	455	283 (62.2%)			
2 or more		172 (37.8%)			
**OBJECTIVE PHYSICAL ENVIRONMENTS (COMPOSITE SCORES)**					
Walk Score (0–100)	455	44.03 (23.73)	Bike Score (0–100)	453	59.13 (20.29)
		0–92			2–99
Transit Score (0–100)	455	35.45 (15.47)			
		0–69			

a *Four-point Likert scale recoding: yes = somewhat disagree + somewhat agree + strongly agree, no = strongly disagree*.

Our final sample was shown to be generally representative of Austin's older populations based on the key demographic characteristic factors and the overall geographic distribution. However, it had an over-representation of females and highly educated people, which may be attributable to the length and content of our survey questionnaire. The paper and online survey participants also showed significant differences in several demographic characteristics, but the binary variable capturing paper vs. online surveys tested in the base models were not significant.

### Social Patterns and Places

**Figure 3:**
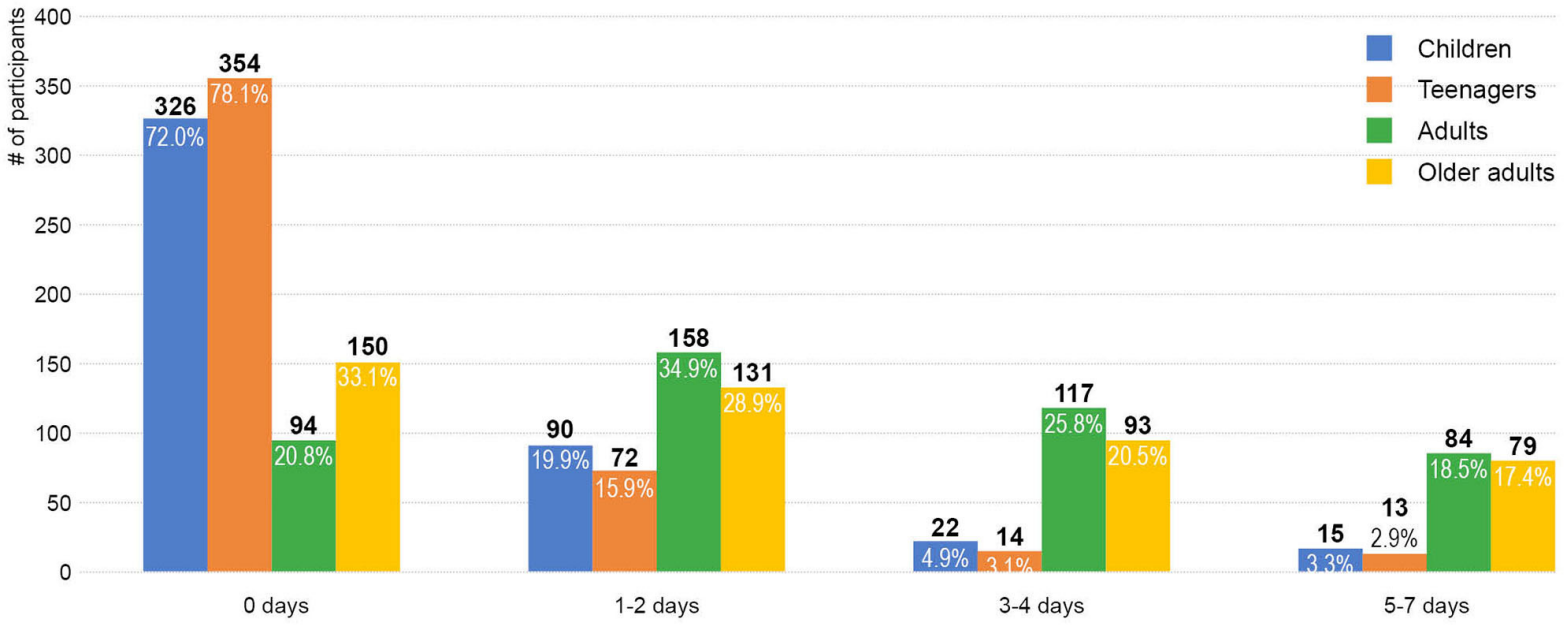
Days of intergenerational and other social activities in a typical week in the neighborhood.

Older adults' social interactions with people of different ages in the neighborhood varied dramatically in frequency. Figure 3 shows that older adults interacted at least once a week with adults (79.2%) and other older adults (66.9%) at much higher rates than with children (28.0%) and teenagers (21.9%).

**Figure 4:**
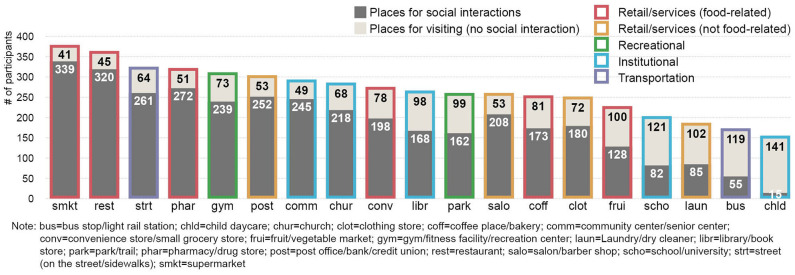
Places for visiting and social interactions at least once a week.

There were a variety of places where participants reported visiting or interacting with others of different ages on a weekly basis (Figure 4). The four most common places for older adults' social interactions were the supermarket, restaurant, street (on the street or sidewalks), and pharmacy/drug store. Additionally, the majority of the study participants interacted with others in three more places, including the gym, fitness facility, or recreation center; post office, bank, or credit union; and community or senior center.

**Figure 5:**
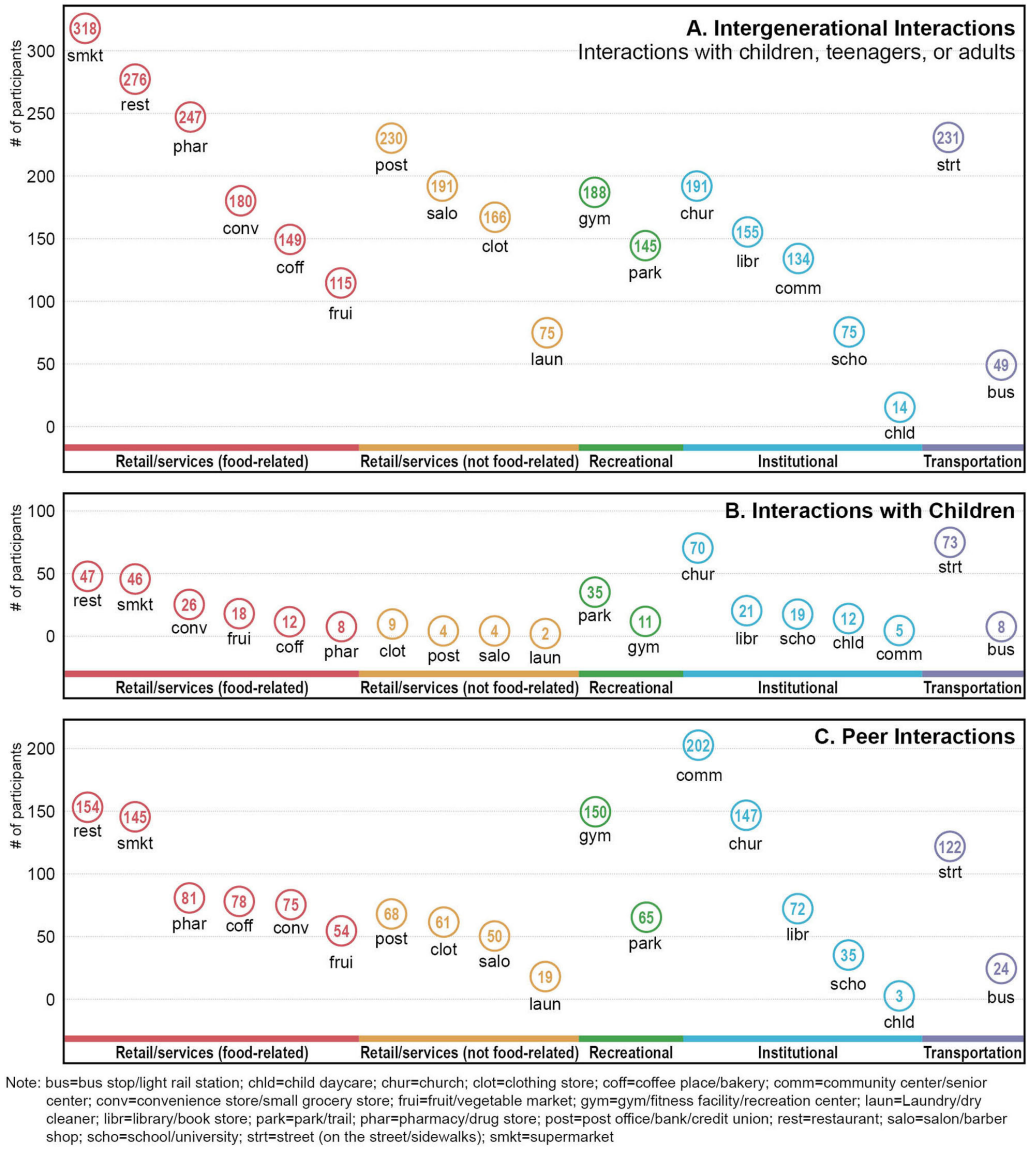
Places for intergenerational and peer interactions at least once a week.

Figure 5 further illustrates popular places used for the three specific types of social activities among older adults, including intergenerational interactions (interactions with children, teenagers, or adults), interactions with children, and peer interactions (interactions with other older adults). Frequently used places for older adults' intergenerational interactions were (1) supermarket, (2) restaurant, (3) pharmacy or drug store, (4) street (on the street or sidewalks), and (5) post office, bank, or credit union. Places popularly used by older adults to engage in peer interactions were (1) community or senior center, (2) restaurant, (3) gym, fitness facility, or recreation center, (4) church, and (5) supermarket. As for social interactions with children, the five most popular places were (1) street (on the street or sidewalks), (2) church, (3) restaurant, (4) supermarket, and (5) park.

### Walking

**Figure 6:**
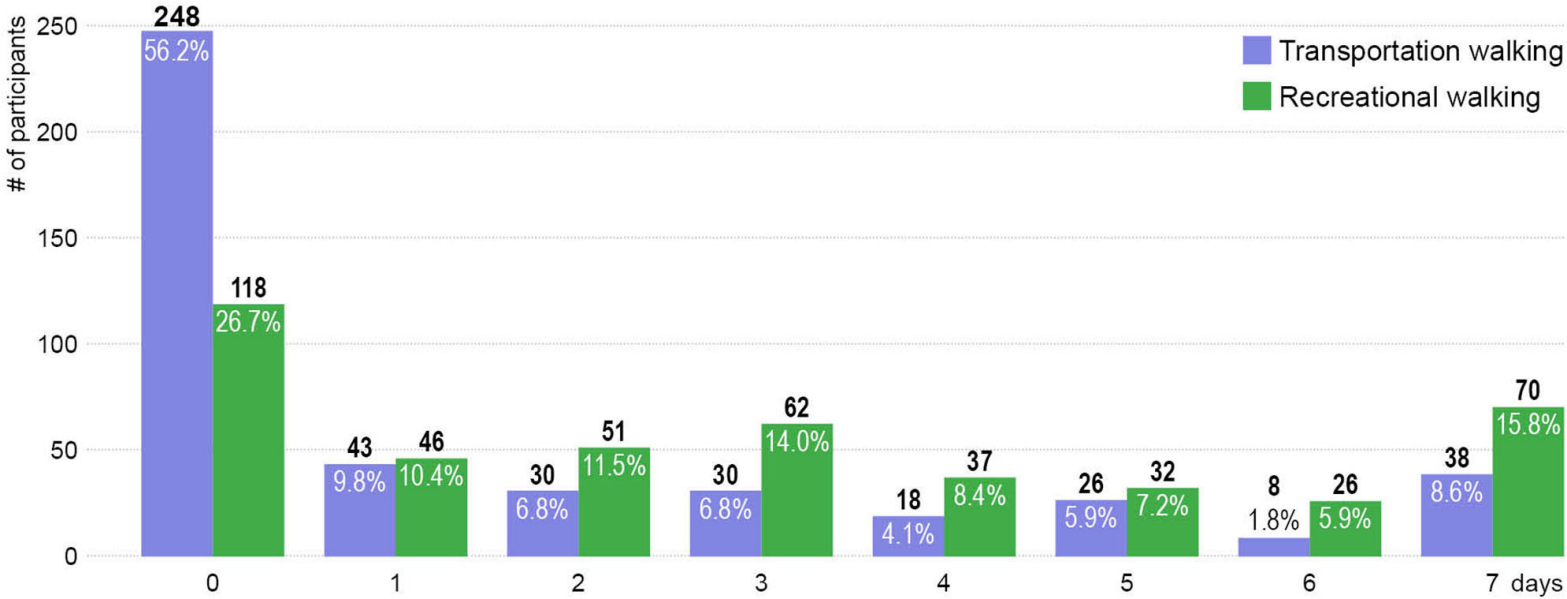
Days of transportation and recreational walking in a typical week.

Recreational walking was more popular for older adults compared to transportation walking (Figure 6). The majority of the participants (73.3%) reported walking for recreation at least once in a typical week, while only 43.8% walked for transportation at least once a week. Moreover, the number of participants (227, 51.4%) who walked for recreation 3+ days in a typical week were almost two times higher than those (120, 27.2%) who walked for transportation 3+ days per week. The two binary walking variables utilized in the bivariate and multivariable regression analyses were transportation walking (43.8% walked vs. 56.2% did not walk at least once a week) and recreational walking (73.3% walked vs. 26.7% did not walk at least once a week).

### Correlates of Intergenerational Interactions and Walking Among Older Adults

#### Perceived Physical Environments

Table 2 summarizes the one-by-one (partially adjusted) model results for the perceived physical environmental variables. Controlled for the base model variables, living in a newly built neighborhood was negatively associated with the odds of interacting with younger generations in the neighborhood (OR = 0.460, *p* = 0.047). Neighborhood walkability was linked with higher odds of interacting with younger generations (OR = 1.461, *p* = 0.013) and being a transportation walker (OR = 1.428, *p* = 0.005). The availability of benches along neighborhood sidewalks was positively correlated with the likelihood of being a recreational walker (OR = 1.966, *p* = 0.024). Neighborhood aesthetics was positively correlated with the likelihood of interacting with children in the neighborhood (OR = 1.401, *p* = 0.023). Traffic safety was linked with lower odds of interacting with younger generations in the neighborhood (OR = 0.676, *p* = 0.009), which might be attributed to neighborhood awareness. For example, older adults who are more socially active may spend more time outdoors in their neighborhood and tend to be more aware of problems like traffic safety issues (e.g., high traffic speeds).

**Table 2:** Perceived environmental correlates of intergenerational interactions vs. walking, from partially adjusted models^#^.

**Variables**	**Intergenerational interactions**				**Walking**			
	**Children**^**a**^		**Intergenerational**^**b**^		**Transportation**^**c**^		**Recreation**^**d**^	
	**OR**	***P*-value**	**OR**	***P*-value**	**OR**	***P*-value**	**OR**	**P-value**
Newly built neighborhood (yes vs. no)			0.460^*^	0.047				
Neighborhood walkability (factor scores; unit: 1)			1.461^*^	0.013	1.428^**^	0.005		
Benches on most of the sidewalks^e^ (yes vs. no)							1.966^*^	0.024
Neighborhood aesthetics (factor scores; unit: 1)	1.401^*^	0.023						
Traffic safety (factor scores; unit: 1)			0.676^**^	0.009				

* *p < 0.05*,

** *p < 0.01, OR: Odds Ratio*.

# *Results from one-by-one tests where physical environmental variables were added to the base models one at a time*.

a *The base model for social interactions with children included nine demographic and socioeconomic variables (i.e., age, gender, race and ethnicity, marital status, education, income, general health conditions, mobility aid, and personal illness) and two residential self-selection variables (i.e., diversity of age groups, social cohesion and support)*.

b *The base model for social interactions with children, teenagers, or adults included eight demographic and socioeconomic variables (i.e., age, gender, race and ethnicity, marital status, education, income, general health conditions, and employment status) and two residential self-selection variables (i.e., diversity of age groups, social cohesion and support)*.

c *The base model for transportation walking included 12 demographic and socioeconomic variables (i.e., age, gender, race and ethnicity, marital status, education, income, general health conditions, housing type, having a dog in the household, employment status, daily sleep time, and mobility aids) and two residential self-selection variables (i.e., neighborhood environments, close to public transportation)*.

d *The base model for recreational walking included 10 demographic and socioeconomic variables (i.e., age, gender, race and ethnicity, marital status, education, income, general health conditions, difficulty walking, having a dog in the household, and employment status), two residential self-selection variables (i.e., neighborhood environments, close to public transportation), and one recruitment channel variable (i.e., recruited from social media)*.

e *Four-point Likert scale recoding: yes = somewhat disagree + somewhat agree + strongly agree, no = strongly disagree*.

#### Objective Physical Environments

Objectively measured physical environments are also important for promoting or hindering older adults' intergenerational interactions and/or walking. Table 3 shows a total number of 37 objectively measured physical environmental variables significantly associated with one or two of the outcomes. These environmental variables contained domains of transportation infrastructure (16, 43.2%), land uses (11, 29.7%), land covers (2, 5.4%), population densities and development activities (5, 13.5%), and composite scores (3, 8.1%). Furthermore, most of the environmental variables were correlated with older adults' intergenerational interactions (29, 78.4%), while significantly fewer environmental variables were associated with older adults' interactions with children (9, 24.3%), transportation walking (13, 35.1%), and recreational walking (2, 5.4%).

**Table 3:** Objective environmental correlates of intergenerational interactions vs. walking, from partially adjusted models^#^.

**Variables**	**Intergenerational Interactions**				**Walking**			
	**Children**^**a**^		**Intergenerational**^**b**^		**Transportation**^**c**^		**Recreation**^**d**^	
	**OR**	***P*-value**	**OR**	***P*-value**	**OR**	***P*-value**	**OR**	***P*-value**
**TRANSPORTATION**								
***Streets and sidewalks***								
Street length (miles; unit: 1)			1.181^**^	**0.001**	1.112^**^	**0.006**		
Sidewalk length (miles; unit: 1)			1.094^**^	**0.002**	1.061^*^	**0.010**		
***Street connectivity***								
Number of Intersections with 3 or more ways (*n*; unit: 10)			1.247^**^	**0.001**	1.169^**^	**0.001**		
Density of intersections with 3 or more ways (n/acre; unit: 1)			1.212^*^	**0.048**	1.240^*^	**0.012**		
***Stop signs***								
Number of stop signs (*n*; unit: 10)			1.226^***^	**0.001**				
Density of stop signs (n/acre; unit: 1)			1.183^**^	**0.003**				
Number of intersections with stop signs (*n*; unit: 10)			1.398^***^	**0.001**	1.144^*^	**0.038**		
Percentage of intersections with stop signs (%; unit: 1%)			6.327^**^	**0.006**				
***Traffic speed***								
Length of high-speed streets (>30 mph) (miles; unit: 1)			1.267^*^	**0.013**				
Percentage of high-speed streets (>30 mph) (%; unit: 10%)	0.797^**^	**0.002**						
***Transit stops***								
Number of transit stops (*n*): 1–5 (vs. 0)			1.010	0.977				
6–10 (vs. 0)			1.927	0.107				
11 or more (vs. 0)			3.271^**^	**0.007**				
Density of transit stops (≥10/100 acres vs. <10/100 acres)			2.592^*^	**0.013**			2.165^*^	**0.024**
Proximity to the closest transit stop [ln(miles); unit: 1]			0.749^*^	**0.027**				
Proximity to the closest rail station [ln(miles); unit: 1]			0.495^***^	**0.000**				
Number of total transit routes (n; unit: 1)			1.155^**^	**0.003**				
Number of transit routes at the closest stop (1 route vs. 2 or more routes)			1.817^*^	**0.041**				
**LAND USES**								
***Office land use***								
Area of offices (acres): >0– <1.5 (vs. 0)			1.340	0.360	1.389	0.242		
≥1.5 (vs. 0)			2.216^*^	**0.021**	2.087^*^	**0.010**		
Percentage of offices (%): >0%– <2% (vs. 0%)			1.476	0.198	1.536	0.104		
≥2% (vs. 0%)			2.300^*^	**0.034**	2.105^*^	**0.020**		
***Food stores***								
Presence of food stores (yes vs. no)			2.299^**^	**0.009**				
Proximity to the closest food store (miles; unit: 1)			0.606^*^	**0.046**	0.576^*^	**0.037**		
***Religious destinations***								
Presence of religious destinations (yes vs. no)			2.180^**^	**0.008**	1.587^*^	**0.045**		
***Recreational destinations***								
Presence of sports and fitness destinations (yes vs. no)	1.834^*^	**0.023**						
Presence of greenbelts (yes vs. no)	0.561^*^	**0.022**			0.580^*^	**0.018**		
Number of parks, excluding natural preserved and greenbelt types (n; unit: 1)	1.134^*^	**0.033**			1.118^*^	**0.049**		
Presence of trails in parks (yes vs. no)			1.888^*^	**0.041**	1.885^**^	**0.007**		
Proximity to the closest park with/next to a water body [ln(miles); unit: 1]	0.803^*^	**0.030**	0.644^***^	**0.001**				
***Undesirable destinations***								
Presence of locally undesirable destinations (yes vs. no)			2.425^**^	**0.003**				
**LAND COVERS**								
Area of tree canopies (acres; unit: 10)			1.133^*^	**0.044**				
Presence of water bodies (yes vs. no)	2.604^*^	**0.010**						
**POPULATION DENSITIES AND DEVELOPMENT ACTIVITIES**								
Net population density (n/acre; unit: 1)			1.038^*^	**0.038**	1.029^*^	**0.036**		
Gross population density (n/acre; unit: 1)							0.928^*^	**0.044**
Number of all development permits issued in 2019 [ln(*n*); unit: 1]	1.243^*^	**0.011**	1.225^*^	**0.035**				
Commercial permits issued in 2019 (yes vs. no)	1.819^*^	**0.016**						
Residential permits issued in 2019 (yes vs. no)	2.195^*^	**0.015**						
**COMPOSITE SCORES**								
Walk Score (scores; unit: 10)			1.171^**^	**0.009**				
Transit Score (scores; unit: 10)			1.290^**^	**0.003**				
Bike Score (scores; unit: 10)			1.213^**^	**0.006**				

* *p < 0.05*,

** *p < 0.01*,

*** *p < 0.001, OR: Odds Ratio*.

*Significant (p < 0.05) correlations are highlighted in bold*.

# *Results from one-by-one tests where physical environmental variables were added to the base models one at a time*.

a *The base model for social interactions with children included nine demographic and socioeconomic variables (i.e., age, gender, race and ethnicity, marital status, education, income, general health conditions, mobility aid, and personal illness) and two residential self-selection variables (i.e., diversity of age groups, social cohesion and support)*.

b *The base model for social interactions with children, teenagers, or adults included eight demographic and socioeconomic variables (i.e., age, gender, race and ethnicity, marital status, education, income, general health conditions, and employment status) and two residential self-selection variables (i.e., diversity of age groups, social cohesion and support)*.

c *The base model for transportation walking included 12 demographic and socioeconomic variables (i.e., age, gender, race and ethnicity, marital status, education, income, general health conditions, housing type, having a dog in the household, employment status, daily sleep time, and mobility aids) and two residential self-selection variables (i.e., neighborhood environments, close to public transportation)*.

d *The base model for recreational walking included 10 demographic and socioeconomic variables (i.e., age, gender, race and ethnicity, marital status, education, income, general health conditions, difficulty walking, having a dog in the household, and employment status), two residential self-selection variables (i.e., neighborhood environments, close to public transportation), and one recruitment channel variable (i.e., recruited from social media)*.

##### Transportation

Our study suggested that neighborhood *streets and sidewalks* and *street connectivity* were significant correlates of older adults' intergenerational interactions and transportation walking. The street length was linked with higher odds of participating in intergenerational interactions in the neighborhood (OR = 1.181, *p* = 0.001) and being a transportation walker (OR = 1.112, *p* = 0.006). The sidewalk length was also positively associated with engaging in intergenerational interactions in the neighborhood (OR = 1.094, *p* = 0.002) and being a transportation walker (OR = 1.061, *p* = 0.010). Two street connectivity variables, the number and density of street intersections, were also positively correlated with intergenerational interactions and transportation walking.

Measures of *stop signs* capturing crossing safety in the neighborhood were significant correlates of older adults' intergenerational interactions and transportation walking. The number of intersections with stop signs were positively associated with engaging in intergenerational interactions (OR = 1.398, *p* = 0.001) and being a transportation walker (OR = 1.144, *p* = 0.038). Another three measures, including the number and density of stop signs and the percentage of intersections with stop signs, were positively correlated with the odds of interacting with younger generations only.

Two variables measuring the *traffic speed* showed different correlations with older adults' social interactions with people of different age groups. The length of high-speed streets was positively associated with the likelihood of interacting with children, teenagers, or adults in the neighborhood (OR = 1.267, *p* = 0.013), while the percentage of high-speed streets was linked with lower odds of interacting with children in the neighborhood (OR = 0.797, *p* = 0.002).

Among the six measures of *transit stops*, the density of transit stops showed positive associations with participating in intergenerational interactions in the neighborhood (OR = 2.592, *p* = 0.013) and being a recreational walker (OR = 2.165, *p* = 0.024). However, the other five measures were positively correlated with intergenerational interactions only: the number of transit stops, the number of total transit routes, the number of transit routes at the closest stop, proximity to the closest transit stop, and proximity to the closest rail station. For the proximity variables in this study, OR < 1 is considered as having a “positive” correlation with the outcomes as a shorter distance means closer proximity and higher accessibility.

##### Land Uses

Among the 12 general (aggregated) land use variables (e.g., residential, recreational) tested in this study, two variables capturing the *office land use* were significantly correlated with older adults' intergenerational interactions and transportation walking. Older adults who had 1.5+ acres of the office land use in the neighborhood had more than twice the odds of interacting with younger generations in the neighborhood (OR = 2.216, *p* = 0.021) and being a transportation walker (OR = 2.087, *p* = 0.010) than those lacking the office land use in the neighborhood. The percentage of the office land use (2+% vs. 0% in the buffer) was also positively correlated with intergenerational interactions (OR = 2.300, *p* = 0.034) and transportation walking (OR = 2.105, *p* = 0.020).

In terms of the destination land use variables, nine variables showed significant associations with one or both outcomes. Older adults living in the neighborhood with *food stores* (i.e., supermarkets, grocery stores, convenience stores without gas stations) were ~2.3 times more likely than those without food stores to participate in intergenerational interactions in the neighborhood (*p* = 0.009). Another measure of food stores, the proximity to the closest food store, showed positive associations with both intergenerational interactions and transportation walking. Several types of institutional destinations were also examined in this study, including educational and community destinations (e.g., school), banks and post offices, offices, and religious destinations. However, only the presence of *religious destinations* was linked with higher odds of engaging in intergenerational interactions in the neighborhood (OR = 2.180, *p* = 0.008) and being a transportation walker (OR = 1.587, *p* = 0.045).

Five measures of recreational destinations were significantly correlated with at least one of the outcomes. Older adults living in the neighborhood with *sports and fitness destinations* were ~1.8 times more likely than those without sports and fitness destinations to interact with children in the neighborhood (*p* = 0.023). The presence of *greenbelts* was negatively associated with the likelihood of interacting with children (OR = 0.561, *p* = 0.022) and being a transportation walker (OR = 0.580, *p* = 0.018), likely due to the limited accessibility and amenities in this type of green space. However, each additional *park* (excluding natural preserved and greenbelt types) in the neighborhood was associated with 13.4% and 11.8% increases in the odds of interacting with children (*p* = 0.033) and being a transportation walker (*p* = 0.049), respectively. Furthermore, the presence of *trails in parks* was linked with higher odds of engaging in intergenerational interactions (OR = 1.888, *p* = 0.041) and being a transportation walker (OR = 1.885, *p* = 0.007). The proximity to the *closest park with/next to a water body* was also positively correlated with both social interactions with children and intergenerational interactions.

Finally, we also examined potential negative roles of undesirable destinations, which included manufacturing (1018, 95.0%), electric generating (28, 2.6%), and warehousing and storage facilities (26, 2.4%) reflecting local land use conditions in Austin, Texas. However, our study indicated that such *locally undesirable destinations* were positively associated with the likelihood of interacting with younger generations in the neighborhood (OR = 2.425, *p* = 0.003).

##### Land Covers

Two types of land covers, *tree canopies* and *water bodies*, showed significant correlations with older adults' intergenerational interactions. For example, each 10-acre increase of tree canopies in the neighborhood was linked with a 13.3% increase in the odds of participating in intergenerational interactions in the neighborhood (*p* = 0.044). Older adults living in the neighborhood with water bodies (e.g., lakes, rivers, ponds) were ~2.6 times as likely as those without water bodies to interact with children in the neighborhood (*p* = 0.010).

##### Population Densities and Development Activities

In terms of population densities, the *net population density* was linked with higher odds of engaging in intergenerational interactions (OR = 1.038, *p* = 0.038) and being a transportation walker (OR = 1.029, *p* = 0.036). The *gross population density* was linked only with a lower likelihood of being a recreational walker (OR = 0.928, *p* = 0.044).

This study also suggested positive associations between property development activities (captured with a proxy measure of development permits issued) and older adults' intergenerational interactions in the neighborhood. Specifically, the number of *all development permits* issued in 2019 was positively associated with the likelihood of interacting with children (OR = 1.243, *p* = 0.011) and participating in intergenerational interactions (OR = 1.225, *p* = 0.035) in the neighborhood. Furthermore, older adults living in the neighborhood with one or more *commercial* or *residential permits* issued were ~1.8 (*p* = 0.016) or 2.2 (*p* = 0.015) times more likely to interact with children in the neighborhood, than those living in areas with no development permits issued in 2019.

##### Composite Scores

Walk Score, Transit Score, and Bike Score were associated only with older adults' intergenerational interactions in the neighborhood. Every 10-point increase in Walk Score, Transit Score, and Bike Score was associated with 17.1% (*p* = 0.009), 29.0% (*p* = 0.003), and 21.3% (*p* = 0.006) increases in older adults' intergenerational interactions in the neighborhood, respectively.

#### Synthesis of Similarities and Differences

This section explores similarities and differences in correlates of intergenerational interactions and walking, regardless of their specific type, to facilitate the synthesis and contextualization of the results from multiple models. To guide the development of relevant policy and intervention programs, it is important to understand which neighborhood factors may bring multiple, synergistic benefits to older adults.

*Similarities* between correlates of intergenerational interactions and correlates of walking among older adults are summarized in Table 4. For the subjective measures, neighborhood walkability was positively associated with both intergenerational interactions and walking. In terms of the objective measures, positive predictors of older adults' intergenerational interactions and walking contained three domains: transportation (i.e., street length, sidewalk length, street connectivity, stop signs, and transit stops), land uses (i.e., office land use, food stores, religious destinations, parks excluding natural preserved and greenbelt types, and trails in parks), and population densities (i.e., net population density). Meanwhile, the presence of greenbelts was a negative correlate of both outcomes.

**Table 4:** Consistent correlates of intergenerational interactions and walking.

**Domain**	**Variable**	**Intergenerational interactions**	**Walking**
Neighborhood perceptions	Neighborhood walkability (S)	**+**	**+**
Transportation	Street length (O)	**+**	**+**
	Sidewalk length (O)	**+**	**+**
	Street connectivity (O)	**+**	**+**
	Stop signs (O)	**+**	**+**
	Number of intersections with stop signs		
	Transit stops (O)	**+**	**+**
	Density of transit stops		
Land uses	Office land use (O)	**+**	**+**
	Food stores (O)	**+**	**+**
	Proximity to the closest food store^*^		
	Religious destinations (O)	**+**	**+**
	Greenbelts (O)	**–**	**–**
	Parks, excluding natural preserved and greenbelt types (O)	**+**	**+**
	Trails in parks (O)	**+**	**+**
Population densities	Net population density (O)	**+**	**+**

*(S): Subjective Measures, (O): Objective Measures, **+**: Significant (p < 0.05) Positive Correlates, **–**: Significant (p < 0.05) Negative Correlates*.

* *The odds ratio of < 1 is considered as having a “positive” correlation with the outcomes as a shorter distance means closer proximity and higher accessibility*.

*Differences* were more evident than similarities in terms of the environmental factors associated with older adults' intergenerational interactions vs. walking (Table 5). Most of these variables were significantly associated with intergenerational interactions only, while another two variables (i.e., benches on sidewalks and gross population density) were significant only for walking. For older adults' intergenerational interactions, positive correlates involved domains of neighborhood perception (i.e., neighborhood aesthetics), transportation (i.e., other measures of stop signs and transit stops), land uses (i.e., another measure of food stores, sports, and fitness destinations, locally undesirable destinations, and parks with/next to a water body), land covers (i.e., tree canopies and water bodies), development activities (i.e., development permits), and composite scores (i.e., Walk Score, Transit Score, and Bike Score). Another two negative correlates of older adults' intergenerational interactions were residence in a newly built neighborhood and traffic safety condition of the neighborhood. Furthermore, different measures of traffic speeds showed both negative and positive correlations with older adults' intergenerational interactions depending on the specific age groups.

**Table 5:** Inconsistent correlates of intergenerational interactions and walking.

**Domain**	**Variable**	**Intergenerational interactions**	**Walking**
Neighborhood Perceptions	Newly built neighborhood (S)	**–**	
	Neighborhood aesthetics (S)	**+**	
Transportation	Benches on sidewalks (S)		**+**
	Stop signs (O)	**+**	
	Number of stop signs		
	Density of stop signs		
	Percentage of intersections with stop signs		
	Traffic safety (S)	**–**	
	Traffic speed (O)	**+** **–**	
	Length of high-speed streets		
	Percentage of high-speed streets		
	Transit stops (O)	**+**	
	Number of transit stops		
	Proximity to the closest transit stop^*^		
	Proximity to the closest rail station^*^		
	Number of total transit routes		
	Number of transit routes at the closest stop		
Land Uses	Food stores (O)	**+**	
	Presence of food stores		
	Sports and fitness destinations (O)	**+**	
	Locally undesirable destinations (O)	**+**	
	Parks with/next to a water body^*^ (O)	**+**	
Land Covers	Tree canopies (O)	**+**	
	Water bodies (O)	**+**	
Population Densities and	Gross population density (O)		**–**
Development Activities	Development permits (O)	**+**	
Composite Scores	Walk Score (O)	**+**	
	Transit Score (O)	**+**	
	Bike Score (O)	**+**	

*(S): Subjective Measures, (O): Objective Measures, **+**: Significant (p < 0.05) Positive Correlates, **–**: Significant (p < 0.05) Negative Correlates*.

* *The odds ratio of < 1 is considered as having a “positive” correlation with the outcomes as a shorter distance means closer proximity and higher accessibility*.

## Discussion and Conclusion

This is one of the first studies that explored specific places older adults used for intergenerational interactions. It also identified significant elements and features of their neighborhood physical environments linked with intergenerational interactions and compared similarities and differences in environmental correlates of older adults' intergenerational interactions vs. walking. This study provided evidence supporting the significant roles of neighborhood environments in promoting older adults' intergenerational interactions and walking, which can further contribute to expanding the existing body of knowledge on environments and aging.

Environmental predictors of older adults' social interactions with children only vs. with all younger age groups (intergenerational interactions) in the neighborhood showed fairly inconsistent patterns. More environmental variables were significant in predicting intergenerational interactions (32 environmental predictors) compared to interactions with children (10 environmental predictors). Only two variables (i.e., proximity to the closest park with/next to a water body and development permits issued in 2019) were correlated with both social interactions with children and intergenerational interactions. Future efforts with fully adjusted models examining the influences of neighborhood environments on various types of social interactions among older adults can contribute to a more comprehensive understanding of the similarities and differences in personal and environmental correlates of various social interactions (e.g., intergenerational vs. peer interactions).

For the environmental correlates of intergenerational interactions vs. walking, intergenerational interactions shared many similar correlates with transportation walking, but not with recreational walking. Only three environmental variables (i.e., benches on most of the sidewalks, the transit stop density, and the gross population density) showed significant associations with older adults' recreational walking, of which only one (transit stop density) was also associated with intergenerational interactions.

We also found counter-intuitive results. For example, locally undesirable destinations (i.e., manufacturing, electric generating, and warehousing and storage facilities), generally considered as negative for outdoor activities, were linked with higher odds of engaging in intergenerational interactions in the neighborhood. Further examinations of the relevant GIS data showed that the manufacturing land uses in our study community consisted mainly of small-scale light manufacturing (e.g., winery, music instrument manufacturing, and digital printing) instead of heavy production manufacturing facilities. These light manufacturing facilities tended to be clustered with other major destinations (e.g., supermarkets, grocery stores) shown to support diverse social activities. The Chi-square test further demonstrated that the presence of locally undesirable destinations was positively correlated with the presence of food stores (*x*^2^ = 45.969, df = 1, *p* < 0.001), which were most popularly used for social interactions in this study (Figures 4, 5). Thus, this study suggested that the presence of manufacturing facilities was a proxy for small mixed use and retail centers that might have provided opportunities for social interactions across different generations in Austin, Texas and similar communities in the US. A previous study, although in smaller communities, showed such small-scale, light manufacturing facilities were positively associated with transportation walking (62). Another counter-intuitive finding was the positive association between the length of high-speed streets and older adults' intergenerational interactions. A similar possibility is that the length of high-speed streets may also capture other environmental elements and features that can promote intergenerational interactions, such as population densities, walking/cycling facilities, and family-friendly destinations. Bivariate analyses indicated that the length of high-speed streets was positively correlated with the net (Pearson R = 0.387, *p* < 0.001) and gross (Pearson R = 0.345, *p* < 0.001) population density, the length of sidewalks (Pearson R = 0.589, *p* < 0.001), and the presence of food stores (independent sample *t*-test = −10.290, *p* < 0.001) in the neighborhood. However, the percentage of high-speed streets in the neighborhood was negatively correlated with interactions with children only, which may be attributed to traffic safety concerns that may be more important for children than for older age groups.

Development permits were positively associated with the likelihood of interacting with younger generations in the neighborhood. The permits issued in 2019 in Austin, Texas included 3,652 commercial and 21,155 residential permits. Locations where these permits were issued during the study period suggest many new infill developments and infrastructure improvements, creating vibrant, age-diverse, and socially engaging environments. Further research is needed to better understand how this widely available variable may help capture some of the difficult-to-quantify aspects of the neighborhood characteristics that are potentially meaningful for residents' social and physical activities.

Walk Score, Bike Score, and Transit Score were positively correlated with older adults' intergenerational interactions in the neighborhood. While developed as primarily destination-driven composite measures to estimate the environmental friendliness to support walking, biking, and transit use, respectively, our study showed that these measures were also significant predictors of social interactions. Previous studies have shown that these scores are linked with health behaviors and outcomes (63–65). Given their ease of use and wide availability, these scores have the potential to promote the consideration of physical environmental variables in intergenerational interaction literature that has largely overlooked the roles of physical environments.

### Limitations

This study has five major limits. First, this was a cross-sectional study that generated results predicting correlations only with no ability to draw causality between variables. Second, another source of limitation is residential self-selection. Although it is possible that older adults who are active or prefer activities choose to live in the neighborhood with features supporting intergenerational interactions and walking, relevant variables (i.e., reasons for selecting their current residence) were controlled in all models, which helped address the potential bias. Third, the survey recall bias and potential measurement errors associated with using newly developed questions posed challenges to this study, but the survey was the only feasible way to collect the data from a sufficient number of eligible participants for this research. To maximize the validity and reliability of the survey instrument, most questions were adapted from existing validated questionnaires. The final instrument was developed after a series of pilot tests (i.e., focus group, one-on-one in-depth discussions) to ensure appropriate length, completeness, clarity, and organization of the questionnaire. The test-retest reliability results suggested acceptable levels of reliability and did not suggest serious recall bias. Fourth, the convenience sampling method led to sample bias (e.g., an over-representation of active and healthy older adults). Relevant variables were tested during the modeling process, and those significant ones (e.g., employment status) were retained in the models. However, many of those variables (e.g., diseases, living arrangements) were not significant suggesting that the risk of serious sampling bias is small. Fifth, generalizability of the significant findings from this research is limited to older adults living in Austin, Texas and in similar communities/cities in the US.

### Implications for Future Research, Practice, and Policy

Responding to the major study limitations discussed above, future studies are needed to utilize more rigorous sampling and analytical strategies, apply case-control and pre-post comparisons, and involve additional locations or communities. As this study has relied only on subjective measures of intergenerational interactions and walking, future research involving objective outcome measures can offer more evidence with more accurate measures. Furthermore, given the significant differences we found for different types of intergenerational interactions, more efforts are needed to investigate the influences of neighborhood environments on various types of intergenerational and other social interactions, such as naturally occurring interactions, casual daily interactions, and formal social interactions. These social interactions can also differ by the locations in which they occur; amount of interactions and their health-significant thresholds; quality of interactions considering emotional preference, experience, satisfaction, etc.; specific age groups older adults interact with; and the level of intimacy. Another area needing more efforts is the development of a clear definition of intergenerational community to guide its operationalization in research and practice, contributing to promoting healthy aging in place.

This study offers insights on the environmental strategies to promote routine intergenerational activities among community-dwelling older adults living in urban communities like those in Austin, Texas. Findings from this study provide practical guidelines for policymakers and design professionals to support the development of age-friendly communities that promote intergenerational interactions and healthy aging in place. Moreover, the evidence supporting the relationships between physical environments and older adults' intergenerational interactions and walking can be translated into evidence-based design and policy principles for creating age-friendly or intergenerational communities. These principles may target transportation infrastructure, land uses, land covers, and neighborhood developments.

Additionally, while it is beyond the scope of this study, the current circumstances affected by COVID-19 bring additional challenges to older adults and their ability to engage in intergenerational interactions and physical activities. Due to their high vulnerability to this virus, older adults are more likely to be socially isolated. The situation is even worse for those who are living in long-term care or assisted living facilities because of the current lockdown of the facilities. Future research appears necessary to understand the impacts of pandemics like this on social/physical activities among older adults and identify effective community-level intervention strategies for supporting social/physical activities while ensuring the safety and health of older adults during pandemics such as COVID-19.

## Conclusions

Our study findings suggest that neighborhood physical environments play essential roles in promoting older adults' intergenerational interactions and walking. Given the significant health benefits, promoting intergenerational interactions and walking among older adults should be viewed as a national/global responsibility. Future policymakers, researchers, and professionals should further investigate social and physical environmental facilitators as well as barriers to creating intergenerational communities that can support healthy living of all generations.

## Data Availability Statement

The raw data supporting the conclusions of this article will be made available by the authors, without undue reservation.

## Ethics Statement

The studies involving human participants were reviewed and approved by the Institutional Review Board at Texas A&M University. Written informed consent for participation was not required for this study in accordance with the national legislation and the institutional requirements.

## Author Contributions

SZ collected the subjective and objective data, conducted statistical analyses, and drafted the manuscript. CL guided the data collection, analysis, and manuscript drafting process, and revised the draft manuscript. HL collected the objectively measured environmental data, ran the GIS models, and drafted part of the MATERIALS AND METHODS section of the manuscript. All authors contributed to the article and approved the submitted version.

## Conflict of Interest

The authors declare that the research was conducted in the absence of any commercial or financial relationships that could be construed as a potential conflict of interest.
